# Investigation of prognostic values of immune infiltration and LGMN expression in the microenvironment of osteosarcoma

**DOI:** 10.1007/s12672-024-01123-9

**Published:** 2024-07-09

**Authors:** Hualiang Xu, Dawei Xu, Yinfeng Zheng, Huajun Wang, Aiguo Li, Xiaofei Zheng

**Affiliations:** 1 Department of Orthopedic, Guangzhou Red Cross Hospital of Jinan University, No. 396, Tongfu Middle Road, Haizhu District, Guangzhou, Guangdong People’s Republic of China; 2 Department of Sports Medicine, The First Affiliated Hospital, Guangdong Provincial Key Laboratory of Speed Capability, The Guangzhou Key Laboratory of Precision Orthopedics and Regenerative Medicine, Jinan University, No. 613, Huangpu Avenue West, Tianhe District, Guangzhou, Guangdong People’s Republic of China

**Keywords:** Osteosarcoma, LGMN, Immune infiltration, Bioinformatics, Prognosis, Therapeutic target, Consensus clustering analysis

## Abstract

**Background:**

Osteosarcoma (OS), the most common primary malignant bone tumor, predominantly affects children and young adults and is characterized by high invasiveness and poor prognosis. Despite therapeutic advancements, the survival rate remains suboptimal, indicating an urgent need for novel biomarkers and therapeutic targets. This study aimed to investigate the prognostic significance of LGMN expression and immune cell infiltration in the tumor microenvironment of OS.

**Methods:**

We performed an integrative bioinformatics analysis utilizing the GEO and TARGET-OS databases to identify differentially expressed genes (DEGs) associated with LGMN in OS. We conducted Gene Ontology (GO), Kyoto Encyclopedia of Genes and Genomes (KEGG), and Gene Set Enrichment Analysis (GSEA) to explore the biological pathways and functions. Additionally, we constructed protein–protein interaction (PPI) networks, a competing endogenous RNA (ceRNA) network, and applied the CIBERSORT algorithm to quantify immune cell infiltration. The diagnostic and prognostic values of LGMN were evaluated using the area under the receiver operating characteristic (ROC) curve and Cox regression analysis. Furthermore, we employed Consensus Clustering Analysis to explore the heterogeneity within OS samples based on LGMN expression.

**Results:**

The analysis revealed significant upregulation of LGMN in OS tissues. DEGs were enriched in immune response and antigen processing pathways, suggesting LGMN's role in immune modulation within the TME. The PPI and ceRNA network analyses provided insights into the regulatory mechanisms involving LGMN. Immune cell infiltration analysis indicated a correlation between high LGMN expression and increased abundance of M2 macrophages, implicating an immunosuppressive role. The diagnostic AUC for LGMN was 0.799, demonstrating its potential as a diagnostic biomarker. High LGMN expression correlated with reduced overall survival (OS) and progression-free survival (PFS). Importantly, Consensus Clustering Analysis identified two distinct subtypes of OS, highlighting the heterogeneity and potential for personalized medicine approaches.

**Conclusions:**

Our study underscores the prognostic value of LGMN in osteosarcoma and its potential as a therapeutic target. The identification of LGMN-associated immune cell subsets and the discovery of distinct OS subtypes through Consensus Clustering Analysis provide new avenues for understanding the immunosuppressive TME of OS and may aid in the development of personalized treatment strategies. Further validation in larger cohorts is warranted to confirm these findings.

## Introduction

Osteosarcoma (OS) is one of the most frequently found primary sarcomas of the bone that primarily affects children, adolescents, and young adults [1]. It is the third most common cancer [2] and the second leading cause of mortality among children. It is characterized by high invasiveness, rapid progression, and poor prognosis. It has been established that ~ 20% of its patients can develop distant metastasis at the time of diagnosis [3], which poses a severe threat to public health. The current treatment strategies mainly include neo-adjuvant chemotherapy, surgical resection of the primary tumors and metastatic ones, and enhanced adjuvant chemotherapy after the surgery [4]. Over the past 30 years, in spite of various extensive clinical trials that have significantly improved clinical outcomes by introducing new chemotherapy drugs or adjusting chemotherapy doses, unfortunately, the prognosis of osteosarcoma remains poor [5–7], with just a 5-year survival rate of 65–70% [8]. Metastatic osteosarcoma (mainly found in the lung parenchymal and distal bones) also has a survival rate of only 19–30% [9, 10]. Although some progress has been made in its diagnosis and treatment in recent years, its prognosis is still far from desirable. Overall, novel strategies for the diagnosis and better prognosis of osteosarcoma are urgently needed.

In recent years, immunotherapy has emerged as a promising therapeutic strategy against different cancers such as breast cancer, lung cancer, and hepatocellular cancer [11, 12]. However, one of the hindrances associated with immunotherapy is the tumor microenvironment (TME), which not only exacerbates the tumor development and metastasis but also is also involved in the resistance to chemotherapy and immunotherapy [13]. TME contains the stromal cells and immune cells that can facilitate the development of cancer and influence the response of the body to tumor therapy, according to a prior report [14]. This finding suggested that TME could play a critical role in the pathogenesis and development of osteosarcoma [15]. In TME, tumor-infiltrating immune cells (TIICs) are also associated with the progression and prognosis of osteosarcoma [16]. TIICs can also differentiate themselves into tumor-associated macrophages (TAMs) and tumor-infiltrating dendritic cells (TIDCs) which can promote both the tumor growth and metastasis [17]. Therefore, it is of paramount importance to systematically evaluate the immune properties of TME and to determine the distribution and function of TAMs in improving the efficacy of immunotherapy.

LGMN, alias Legumain, is a subtype of the family of cysteine protease c13 [18] that can specifically hydrolyze the aspartate peptide bond [19]. Although minimally expressed in normal tissues and rarely in tissues near cancer [20], LGMN is usually overexpressed in the microenvironments of the various solid tumors [21] as breast cancer, colorectal cancer, ovarian cancer, etc. Extensive studies have shown that as LGMN is closely related to the tumor metastasis, diffusion, and invasion, and considered as a potential biomarker indicative of poor prognosis [22]. However, apart from this knowledge about it, there are only few reports on how LGMN is involved in the expression level of human osteosarcoma cells and in the development of human osteosarcoma. Therefore, we aimed to decipher the possible role of LGMN in TAMs in the progression and metastasis of osteosarcoma.

Against this background, we have conducted this study to investigate the clinical and prognostic significance of LGMN to TAMs in osteosarcoma. In this study, we have investigated LGMN expression in osteosarcoma and normal tissues based on the GEO and the database. LGMN expression was then systematically integrated and analyzed by using various methods and databases. The main objective of this study was to provide novel insights into the molecular mechanisms that can contribute to the development of osteosarcoma. This study was also designed to validate the hypothesis that LGMN might work as a potential diagnostic and prognostic biomarker and/or therapeutic target of osteosarcoma.

## Materials and methods

### Sample sources and the screening of differential (co-expression) genes

Given the significant difference in LGMN in the GSE42352 dataset between 15 normal samples and 103 osteosarcoma samples, we first grouped the median value of LGMN expression of the 103 osteosarcoma samples in the GSE42352 dataset. Thereafter, we divided them into a low-expression group of 51 cases and a high-expression group of 52 cases. The comparability of the GSE42352 and TARGET-OS datasets is delineated as follows: (1) Sample Origin: The GSE42352 dataset comprises 15 normal and 103 osteosarcoma samples, sourced from the Gene Expression Omnibus (GEO), presenting a broad spectrum of osteosarcoma transcriptomic profiles. Conversely, the TARGET-OS dataset, furnished by The National Cancer Institute's Therapeutics Applicable Research to Generate Effective Treatments (TARGET) program, is focused on 101 pediatric and adolescent osteosarcoma cases. Collectively, these datasets embody distinct osteosarcoma patient populations. (2) Data Processing: Both datasets have undergone analogous normalization procedures (e.g., RMA normalization) and quality control measures to ascertain data uniformity. (3) Key Features Comparison: We juxtaposed shared clinical characteristics and gene expression profiles in both datasets, affirming congruency in feature distribution, thereby validating their comparability.

Differential analysis was performed using the limma package, where genes were screened based on the fold change (FC) between groups and the significance of difference (P-value). A threshold of |logFC|> 1 and P-value < 0.05 was set, leading to the identification of 86 co-expressed genes. The heatmap and ggplot2 packets were used to visualize the different genes in a heatmap and a volcano plot. The median expression values of the 103 osteosarcoma samples from the TARGET-OS data set (validation set) were grouped and further divided into the low-expression group with 50 cases and a high-expression group of 51 cases. 136 differentially expressed genes (DEGs) were then obtained using the same method. After the two groups of DEGs were intersected, we obtained 26 DEGs in total.

We opted to use the median LGMN expression within tumor samples as the stratification criterion, rather than the expression values from the control group, for the following reasons: Firstly, the variability in LGMN expression within tumor samples better reflects the heterogeneity of osteosarcoma, which is crucial for accurately predicting patient prognosis. Secondly, the median expression value provides a tumor-specific benchmark that aids in identifying high-expression tumor samples associated with adverse outcomes. Additionally, this approach aligns with precedents in the literature and is validated by the ROC curve and survival analyses. For the independent datasets GSE42352 and TARGET-OS, we individually determined the median LGMN expression to categorize samples into high and low expression groups. To compare the consistency of LGMN expression levels across the two datasets, we conducted additional statistical analyses, revealing comparable median expression levels. We believe that this grouping method reflects similar trends in LGMN expression between different datasets, thereby enhancing the credibility of our research outcomes.

### Analysis of GO, KEGG, and GSEA pathway

The cluster Profiler package [23] (FDR < 0.1) was employed for KEGG pathway analysis and GO analysis of the DEGs (GO includes Biological Processed, Molecular Functiond and Cellular Components). The expression matrix of the differential genes grouped by high and low expressions of LGMN was used for GSEA analysis [24], with msigdb. v7.0. entrez. gmt as the selected reference gene set. Then ggPlot2 package was utilized to visualize GO, KEGG, and GSEA pathways by creating the related bar charts, bubble charts, and enrichment maps.

### Construction of PPI molecular interaction network and extraction of the key gene modules

STRING database [25], containing 18,838 human proteins and 25,914,693 core network interactions was used to design the protein–protein interaction (PPI) molecular network of the different genes. The PPI network was then constructed from the cytoscape-identified DEGs. The highest confidence interaction score was set as 0.4. MCODE and cytoHubba plug-ins of Cytoscape software was used to screen and visualize the Hub genes of PPI network modules.

### Correlation between infiltration expression of immune cells and immunophenotypes

CIBERSORT [26] is an algorithm widely used to characterize the cellular composition of the different complex tissues by gene expression values in the solid tumors. LM22 (a special genetic marker) signature algorithm was employed as a special genetic marker, which contains 547 distinct genes. It can distinguish 22 immune cell subtypes downloaded from the CIBERSORT portal (http://cibersort.stanford.edu/). In this study, CIBERSORT package and LM22 algorithm were used to calculate the infiltration abundance of 22 immune cell subtypes in the 103 osteosarcoma samples between the high and low LGMN expression groups containing the different T cells, B cells, plasma cells, natural killer cells, and different myeloid subsets. We also analyzed the correlation of expression distribution of 22 immune cell subtypes between LGMN high and low expression groups. At the same time, a correlation heat map was drawn with red representing the positive correlation, blue representing the negative correlation, where the darker the color, the closer the value is to 1, and the stronger the correlation.

A list of immune-related genes was acquired from the ImmPort database (https://immport.niaid.nih.gov/home) [27]. The DEGs were then intersected with the phenotypic-related genes, and Venn diagrams were drawn using Venn diagram package. Thereafter, the plot function of the R language was used to analyze the correlation between the DEG sets of the high and low LGMN expression groups and that of the phenotypic-related genes. The highly correlated results were finally visualized to draw a correlation heat map.

### Construction of ceRNA network

We used the limma package to obtain a differential lncRNA expression matrix, based upon which prediction about a highly conserved miRNA list of the differentially expressed lncRNAs via the miRcode website was made. Then, a predictive list of miRNA TARGET genes was produced based upon miRDB, miRTarBase, and TARGET Scan databases. The mRNA obtained from the TARGET database was intersected to construct the ceRNA network.

### Evaluation of the diagnostic performance by the area under curve (AUC) of ROC

We used the pROC package for plotting the receiver operating characteristic (ROC) curve. The true positive rate (sensitivity) was plotted in ROC curve analysis against the false positive rate (1-specificity). ROC curve was used to analyze the potential expression level of LGMN and to analyze whether the 15 normal samples could be well distinguished from 103 osteosarcoma samples. Moreover, optimal cut-off value was also determined, which can produce the highest likelihood ratio to judge the recognition threshold of LGMN for osteosarcoma. The risk model was later used to evaluate the possible impact of each biomarker on both survival and prognosis.

The survival ROC package was used to determine the accuracy of the risk model in predicting the survival and prognosis of osteosarcoma. If AUC > 0.5, it was considered to be suitable to reflect the diagnostic value of biomarkers for diseases directly. In addition, the closer the area under the ROC curve was to 1 and to the point (0, 1), the higher the authenticity of the diagnostic test was considered.

### Cox regression and risk model construction

We employed Cox regression for the univariate analysis of the gene matrix downloaded from the Target database. We selected 26 common differential genes as a set of risk factors and extracted their univariate regression results. Further multivariate Cox regression analysis of this gene set was performed to predict the different biomarkers of high-risk osteosarcoma.

### LGMN and risk model mutation analysis

As the results of this study indicated that LGMN could be related to the prognosis of cancer patients, we further analyzed the genetic mutation in great depth. The CBioPortal database was used to analyze the probability distribution of the mutation types, mutation status, and variations in CNVs copy number of each gene in 32 TCGA pan-cancer data sets with 5 gene risk models.

### Survival analysis

Survival package data was used for the target-OS data set survival analysis, including overall survival (OS), progression-free survival (PFS), and time to progression (TTP) to validate the accuracy of the risk model for predicting overall survival prognosis of osteosarcoma.

### Osteosarcoma subtype construction

Consensus Clustering is a resampling-based algorithm designed to identify each member and its subgroup number, and to validate the rationality of clustering. It operates through multiple iterations on subsamples of the dataset, utilizing subsampled-induced variability to provide indicators of clustering stability and parameter decision-making. The consensus clustering method, implemented using the R package ConsensusClusterPlus, is employed to identify different disease subtypes within osteosarcoma samples based on module genes. In this process, the number of clusters is set between 2 and 9, with 50 repetitions drawing 80% of the total samples, with the parameters clusterAlg = "kmeans" and distance = "pearson". Subsequently, a cluster comparison plot is used to validate the expression differences of genes across different disease subtypes.

### Statistical analysis

R Statistics software (Version 4.0.2) was used for the statistical analysis and a P value less than 0.05 considered as statistically significant.

## Results

### Differential expression analysis

**Fig. 1:**
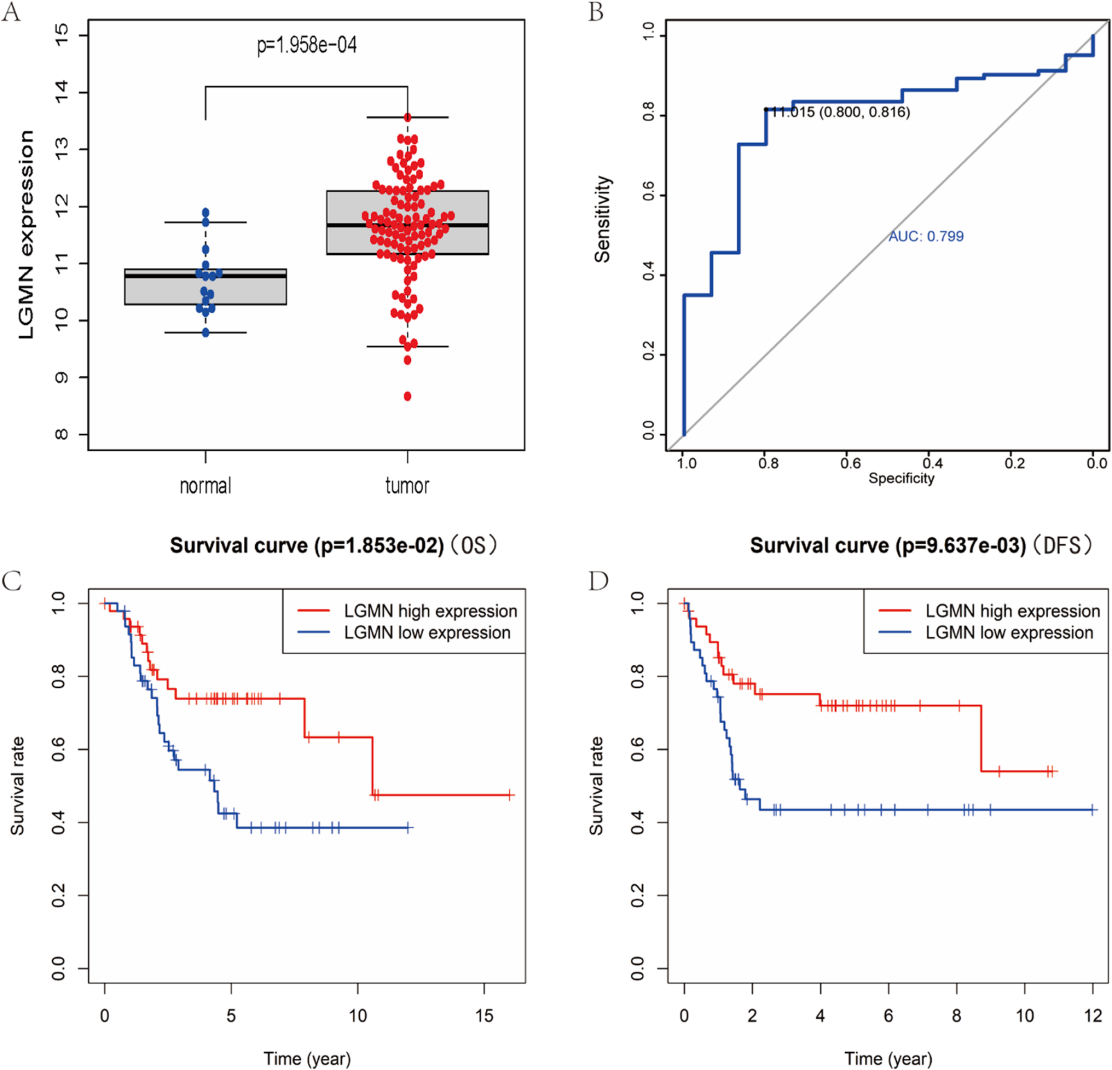
The significance of LGMN expression difference, diagnostic evaluation and the prognostic value in the validation set. **A **The median expression values of LGMN in 103 osteosarcoma samples included in the test set were divided into high and low expression groups. Volcano plot showed the significant difference in DEGs profiles between the two groups. **B** Receiver operating characteristic (ROC) curves and time-dependent area under the curve (AUC) analysis with the diagnostic value of LGMN for osteosarcoma. **C** The effect of LGMN on the overall survival (OS) of osteosarcoma was found to be statistically significant. **D** The effect of LGMN on disease-free survival (DFS) of osteosarcoma was observed to be statistically significant

**Fig. 2:**
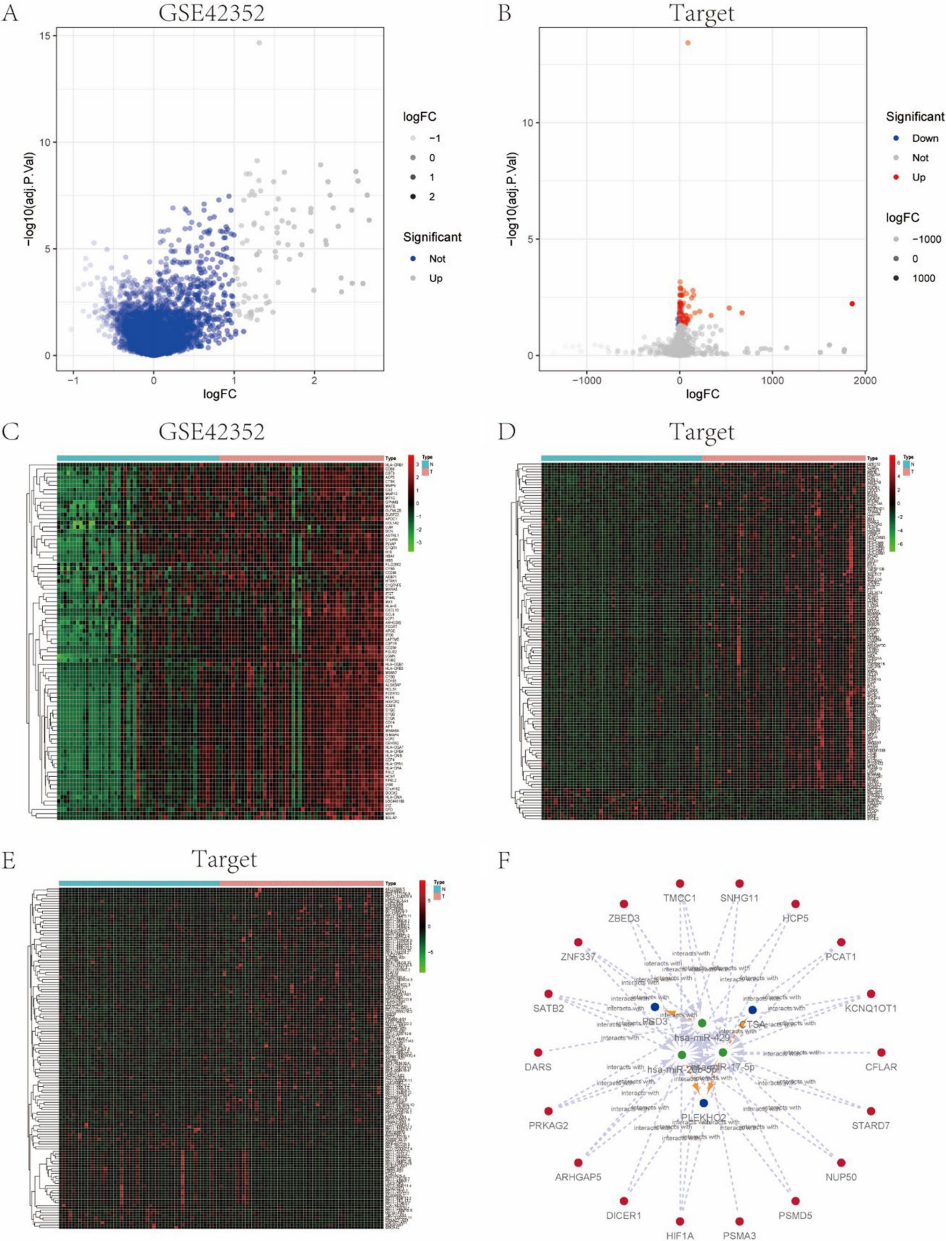
Analysis of differences among single gene and co-expressed gene. **A** The median expression value of LGMN in 103 osteosarcoma samples from the GSE42352 data set (test set) were divided into the high and low groups, and volcano plot indicated the significant difference in DEGs profiles between the two groups. **B** The median expression values of LGMN in 101 osteosarcoma samples from the target-OS data set were divided into the high and low expression groups, and volcano plot showed significant difference in DEGs profiles between the two groups. **C** Significant differences in the genes between the two groups in the GSE42352 data set have been shown as heat plots. **D** The significantly altered DEGs between the two groups have been shown in the form of heat plot in the target-OS data set (validation set). **E** The median expression values of LGMN in the database were divided into high and low expression groups, and lncRNA differences were analyzed for constructing the heat plots. **F** The construction of the ceRNA network. Blue circles represent mRNA, green circles represent miRNA, and red circles represent lncRNA

We utilized the GSE42352 data set from 15 normal samples and 103 osteosarcoma samples as the test set for the differential analysis. Interestingly, the analysis revealed significant differences in LGMN expression between the normal and the osteosarcoma patients (Fig. 1A). Thereafter, we divided the median expression values of LGMN in the 103 tumor samples of the test set into high and low expression groups of 50 and 51 respectively. We employed the LIMMA package for differential analysis and visualized the results by constructing a volcano plot (Fig. 2A) and a heatmap (Fig. 2C). In addition, 101 patients with osteosarcoma from the TARGET database were divided into high and low expression groups of 50 and 51 respectively, using the median expression value of LGMN. We then screened the differential (co-expressed) genes according to the fold change and P-value between the two groups. With |logFC|> 1 and P value < 0.05 as the threshold, 86 co-expressed genes were identified. Moreover, we used heatmap and ggplot2 packages to visualize the heatmap and volcano plot of different genes (Fig. 2B, D). The median expression values in 101 osteosarcoma samples from the target-OS data set (validation set) were grouped into high and low expression groups of 50 and 51 respectively. We next obtained 136 DEGs using the same method (Fig. 1B, D). Thereafter, upon intersecting the two DEGs groups, we obtained 26 common DEGs (Fig. 2E, F).

### Pathway analysis of GO, KEGG, and GSEA

**Fig. 3:**
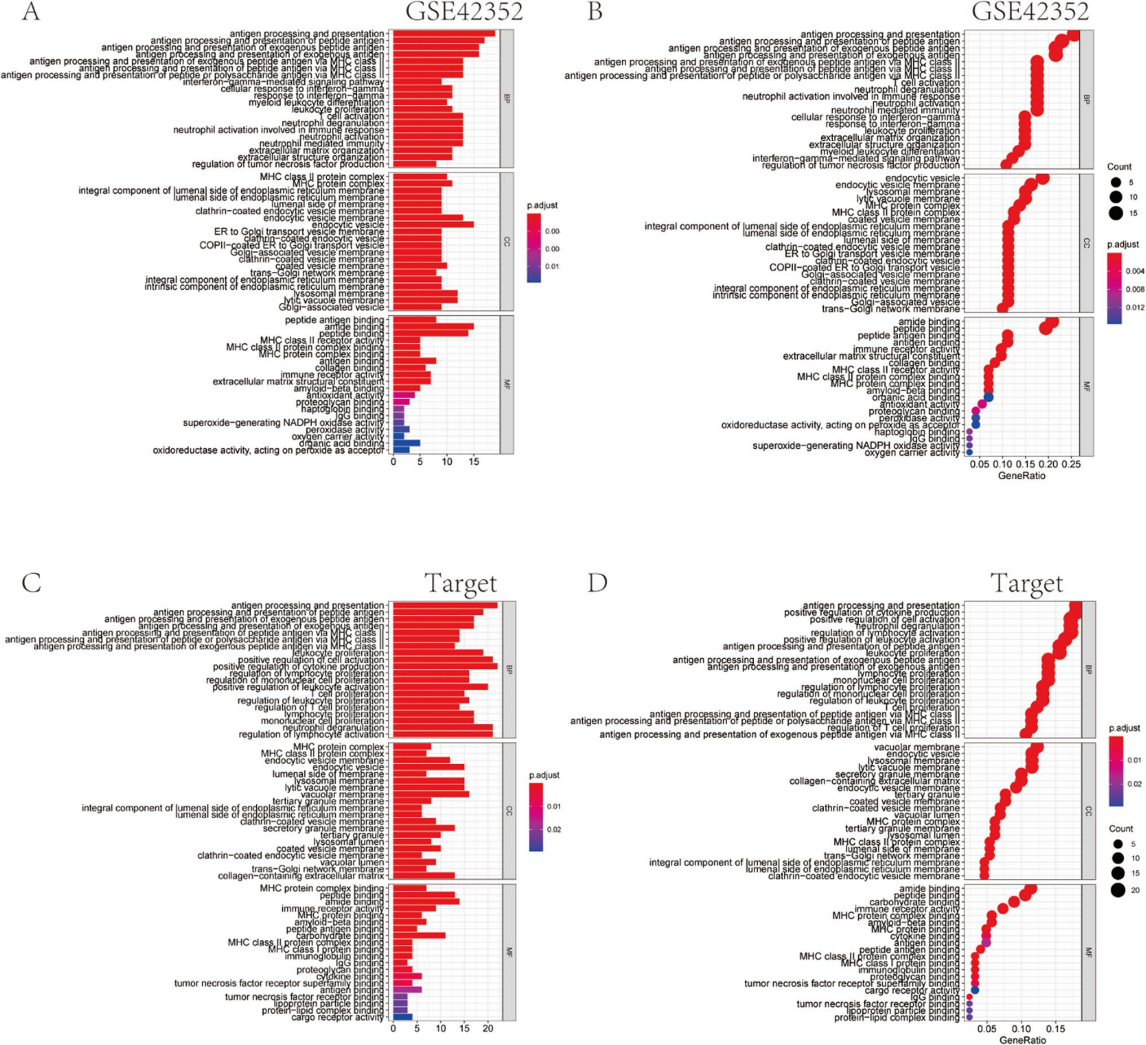
GO pathway enrichment analyses. **A** The bar chart shows the GO pathway enrichment analysis of GSE42352 DEGs, with the length representing the gene enrichment degree and color representing significance. **B** Bubble plot manifests GO pathway enrichment analysis of GSE42352 DEGs. Bubble size represents the degree of the gene enrichment, and color represents significance. **C** The bar chart stands for GO pathway enrichment analyses of the database different-gene, with length representing the degree of gene enrichment and color indicating the significance. **D** The bubble diagram illustrates GO pathway enrichment analysis of TARGET database. The bubble size represents the degree of gene enrichment and color indicates the significance

**Fig. 4:**
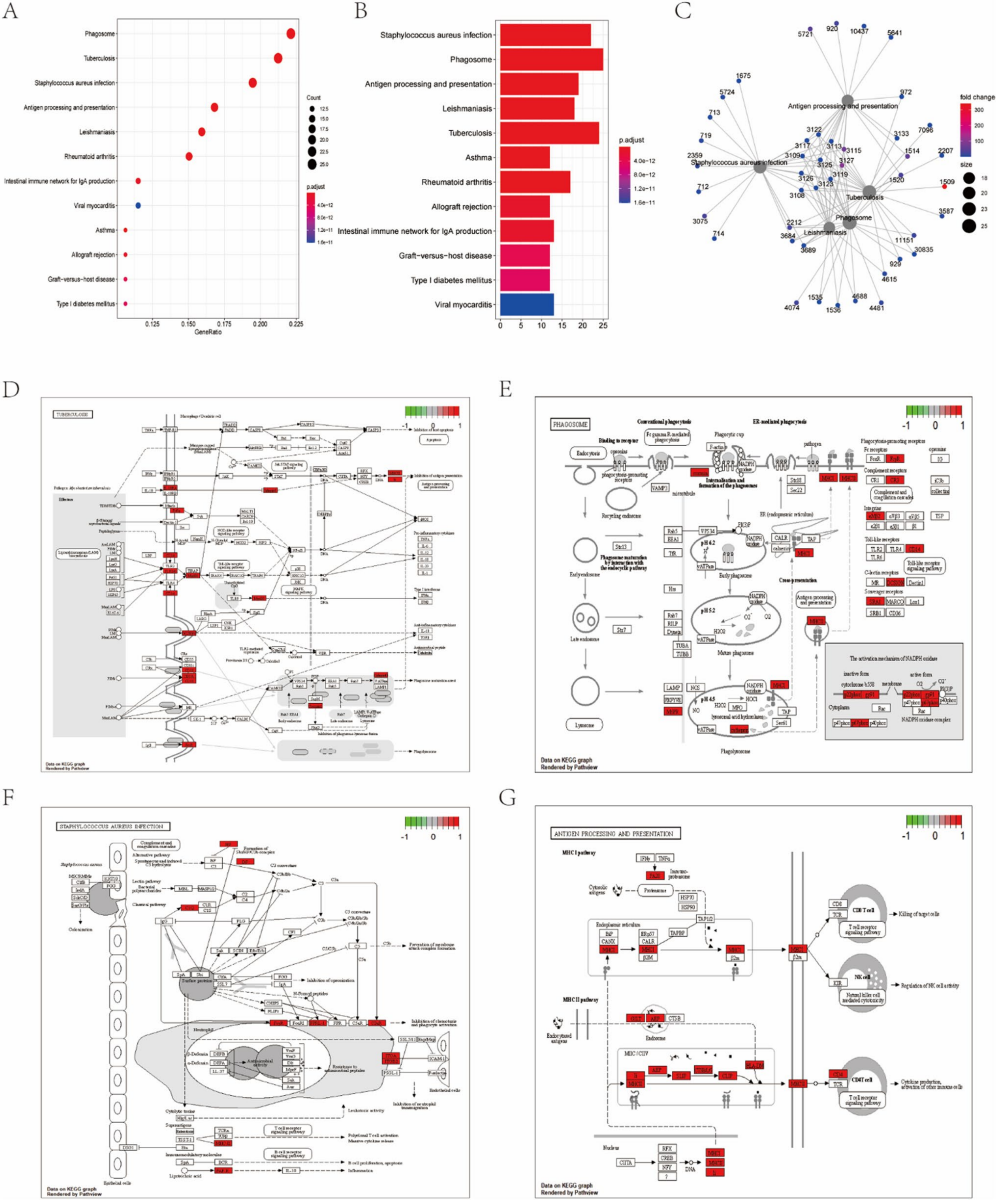
KEGG pathway analyses. **A** GSE42352 data set DEGs clusterProfiler package KEGG pathway analyses have been shown in bubble diagram, with bubble size representing gene enrichment number, color gradually ranging from blue to red indicating the gradual increase in the significance. **B** GSE42352 data set DEGs clusterProfiler package KEGG pathway analyses have been shown in bar chart, with the length representing the gene enrichment degree and color ranging from blue to red indicating a gradual increase in significance. **C** GSE42352 data set DEGs clusterProfiler package KEGG analysis of the enrichment diagram which depicts the relationship between gene ID and the pathway. **D** Type I diabetes mellitus pathway plot. **E** Rheumatoid arthritis pathway plot. **F** Influenza A pathway plot. **G** Staphylococcus aureus infection pathway plot

The bar graph, bubble graph, and enrichment graph of the cluster Profiler package for GO pathway enrichment analysis (Fig. 3; Table 1) and KEGG pathway analysis have been presented (Fig. 4; Table 2). The length of the bar and the size of the bubble represent the degree of Gene enrichment. The gradual color ranging from blue to red indicates the gradual increase in the significance. The two enrichment methods consistently showed four distinct pathways (Fig. 3A–D), including tuberculosis-related, phagosome-related, staph infection-related, antigen processing, and presentation pathways. These results suggested that LGMN was associated with infection immunity, which might influence the body's immune defense system and indirectly can affect osteosarcoma's development and prognosis.

**Table 1:** GO pathway analyses list through the clusterProfiler package

Ontology	ID	Description	p.adjust	Count
BP	GO:0019882	Antigen processing and presentation	3.79E−17	19
BP	GO:0048002	Antigen processing and presentation of peptide antigen	7.47E−16	17
BP	GO:0002478	Antigen processing and presentation of exogenous peptide antigen	4.49E−15	16
BP	GO:0019884	Antigen processing and presentation of exogenous antigen	6.36E−15	16
BP	GO:0019886	Antigen processing and presentation of exogenous peptide antigen via MHC class II	2.94E−14	13
BP	GO:0002495	Antigen processing and presentation of peptide antigen via MHC class II	3.61E−14	13
BP	GO:0002504	Antigen processing and presentation of peptide or polysaccharide antigen via MHC class II	3.61E−14	13
BP	GO:0042110	T cell activation	4.29E−06	13
BP	GO:0043312	Neutrophil degranulation	6.69E−06	13
BP	GO:0002283	Neutrophil activation involved in immune response	6.71E−06	13
CC	GO:0030139	Endocytic vesicle	2.36E−11	15
CC	GO:0030666	Endocytic vesicle membrane	3.11E−12	13
CC	GO:0010008	Endosome membrane	4.46E−07	13
CC	GO:0005765	Lysosomal membrane	1.60E−07	12
CC	GO:0098852	Lytic vacuole membrane	1.60E−07	12
CC	GO:0062023	Collagen-containing extracellular matrix	5.51E−07	12
CC	GO:0005774	Vacuolar membrane	6.20E−07	12
CC	GO:0042611	MHC protein complex	8.01E−19	11
CC	GO:0009897	External side of plasma membrane	2.84E−06	11
CC	GO:0042613	MHC class II protein complex	8.01E−19	10
MF	GO:0033218	Amide binding	8.04E−10	15
MF	GO:0042277	Peptide binding	8.04E−10	14
MF	GO:0042605	Peptide antigen binding	7.51E−11	8
MF	GO:0003823	Antigen binding	7.68E−06	8
MF	GO:0140375	Immune receptor activity	1.99E−05	7
MF	GO:0005201	Extracellular matrix structural constituent	8.99E−05	7
MF	GO:0005518	Collagen binding	7.68E−06	6
MF	GO:0032395	MHC class II receptor activity	1.22E−08	5
MF	GO:0023026	MHC class II protein complex binding	1.67E−07	5
MF	GO:0023023	MHC protein complex binding	1.64E−06	5

**Table 2:** KEGG pathway analyses list through the clusterProfiler package

ID	Description	Count	p.adjust	qvalue
hsa04145	Phagosome	25	1.58E−18	1.22E−18
hsa05152	Tuberculosis	24	6.36E−16	4.92E−16
hsa05150	Staphylococcus aureus infection	22	3.70E−19	2.86E−19
hsa04612	Antigen processing and presentation	19	2.34E−17	1.81E−17
hsa05140	Leishmaniasis	18	3.61E−16	2.79E−16
hsa05323	Rheumatoid arthritis	17	1.50E−13	1.16E−13
hsa04514	Cell adhesion molecules	16	2.13E−09	1.65E−09
hsa05164	Influenza A	16	1.39E−08	1.08E−08
hsa05169	Epstein-Barr virus infection	16	1.35E−07	1.04E−07
hsa05168	Herpes simplex virus 1 infection	16	0.0078	0.0060

**Fig. 5:**
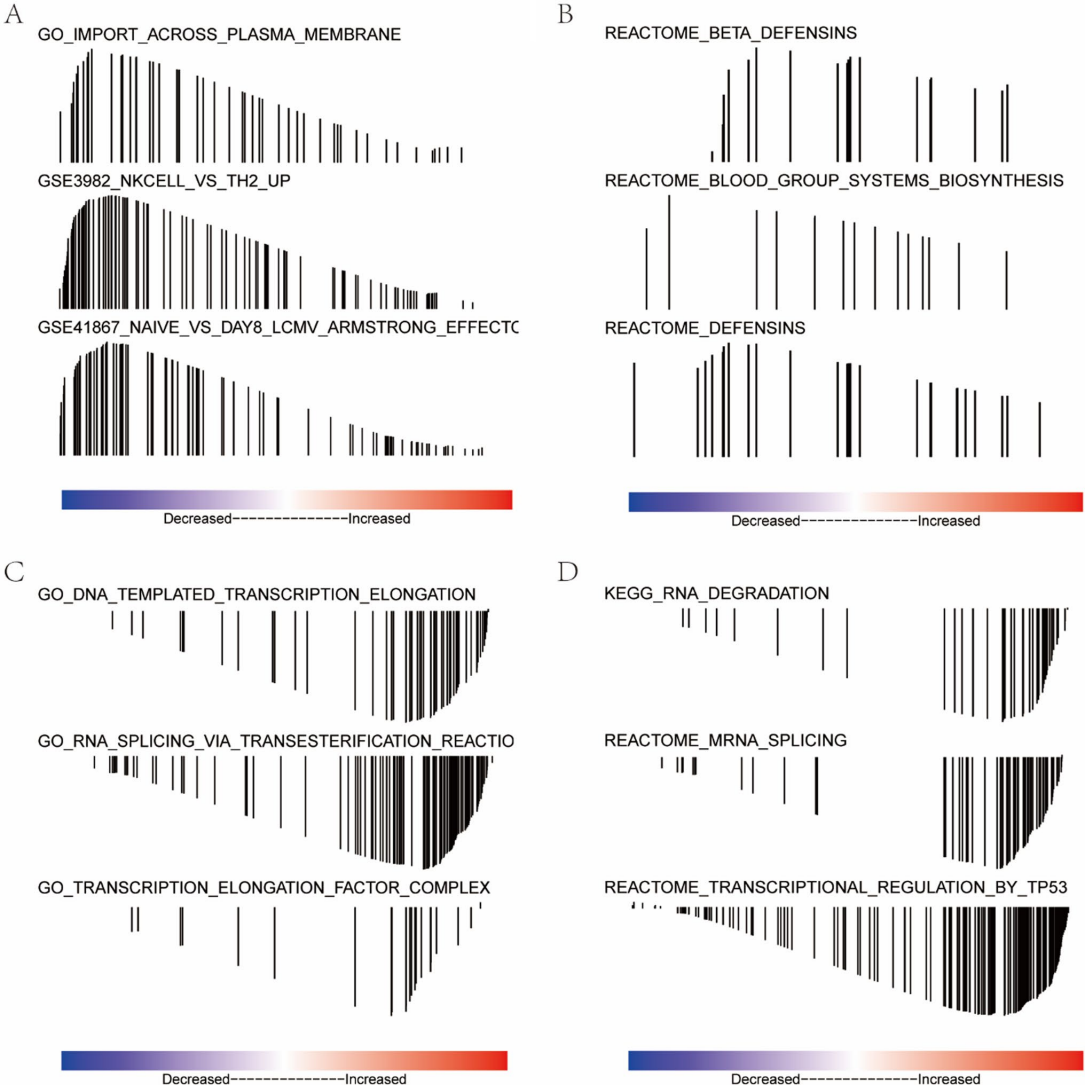
GSEA pathway analyses. GSEA software was employed for GO pathway enrichment analysis, and to determine the expression matrices of DEGs were grouped into LGMN high and low expression groups. **A** Functional correlation enrichment in LGMN high expression group. **B** Reactome pathway correlation enrichment in LGMN high expression group. **C** Functional correlation enrichment in LGMN low expression group. **D** Correlation enrichment of KEGG and Reactome database pathways in LGMN low expression group

Further, we conducted GO pathway enrichment analysis using GSEA software in Java environment. The “msigdb.v7.0.entrez.gmt” was selected as the reference gene set for expression matrix of DEGs grouped based on the high and low LGMN expression groups. We found that translocation and cell membranes of various immune cells were enriched in the high LGMN expression group (Fig. 5A). Moreover, biosynthesis of the various related pathway defensins, β—defensins and blood systems were enriched by the Reactome database in the high LGMN expression group (Fig. 5B). In addition, function-related enrichment in the low LGMN expression group included DNA template transcriptional extension, RNA splicing via transcriptional reaction and transcriptional elongation factor complex (Fig. 5C). KEGG and Reactome databases in the low LGMN expression group enriched the following pathways, including RNA degradation, transcriptional regulation of TP53, mRNA splicing, etc. (Fig. 5D). These results suggested that as the high expression of LGMN could be related to the cancer and immune microenvironment, it might indirectly affect the occurrence of cancer through various mechanisms such as cytokines and metabolic enzymes related to the above pathways or phenotypes. Given this consideration, we next used the RColorBrewer package to visualize the GSEA collection.

### PPI protein interaction network construction and Hub gene screening

**Fig. 6:**
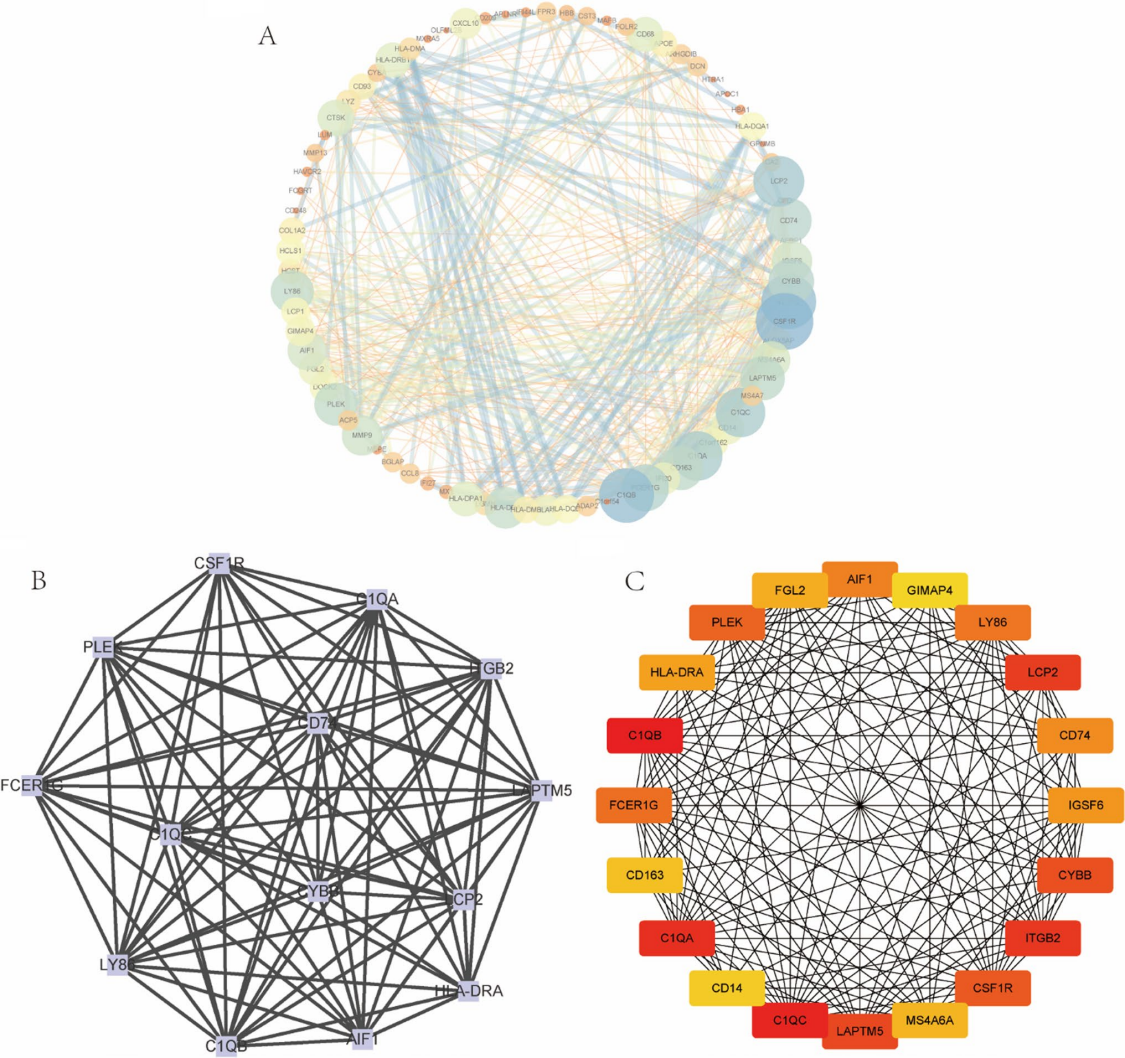
PPI network construction and the module analysis. **A** The figure represents a Protein–Protein Interaction (PPI) network constructed using a molecular interaction list obtained from the STRING database. The size of the circles indicates the number of protein interactions; the larger the circle, the darker the color. **B** The key gene sets were obtained through the MCODE plug-in of Cytoscape software. **C** The MCC algorithm on cytoHubba plug-in of Cytoscape software was used to obtain the Top20 most closely related Hub gene collections

STRING database was employed to construct a novel network for the protein-to-protein interaction among different genes (Fig. 6A). We obtained a bar chart of Top10 closely related to Hub genes using the MCC analysis approach on cytoHubba plug-ins in Cytoscape based on the protein interactions predicted from the String database (Fig. 6B). The cytoHubba plug-in was displayed to analyze the Top30 genes on cytoHubba, with the colors ranging from light to dark to indicate the gradual closeness in the relationship (Fig. 6C).

### Construction of ceRNA network

We next employed LIMMA package to obtain the differentially expressed lncRNA matrix from the TARGET database, and generated a list of miRNA results, which were highly conserved with the differentially expressed lncRNA at the miRcode website. Subsequently, a list of miRNA TARGET genes using miRDB, miRTarBase and TargetScan databases was generated. Then we intersected the mRNAs obtained from the TARGET database to construct the ceRNA network (Fig. 2F).

### Correlation between immune infiltration and immunophenotype

**Fig. 7:**
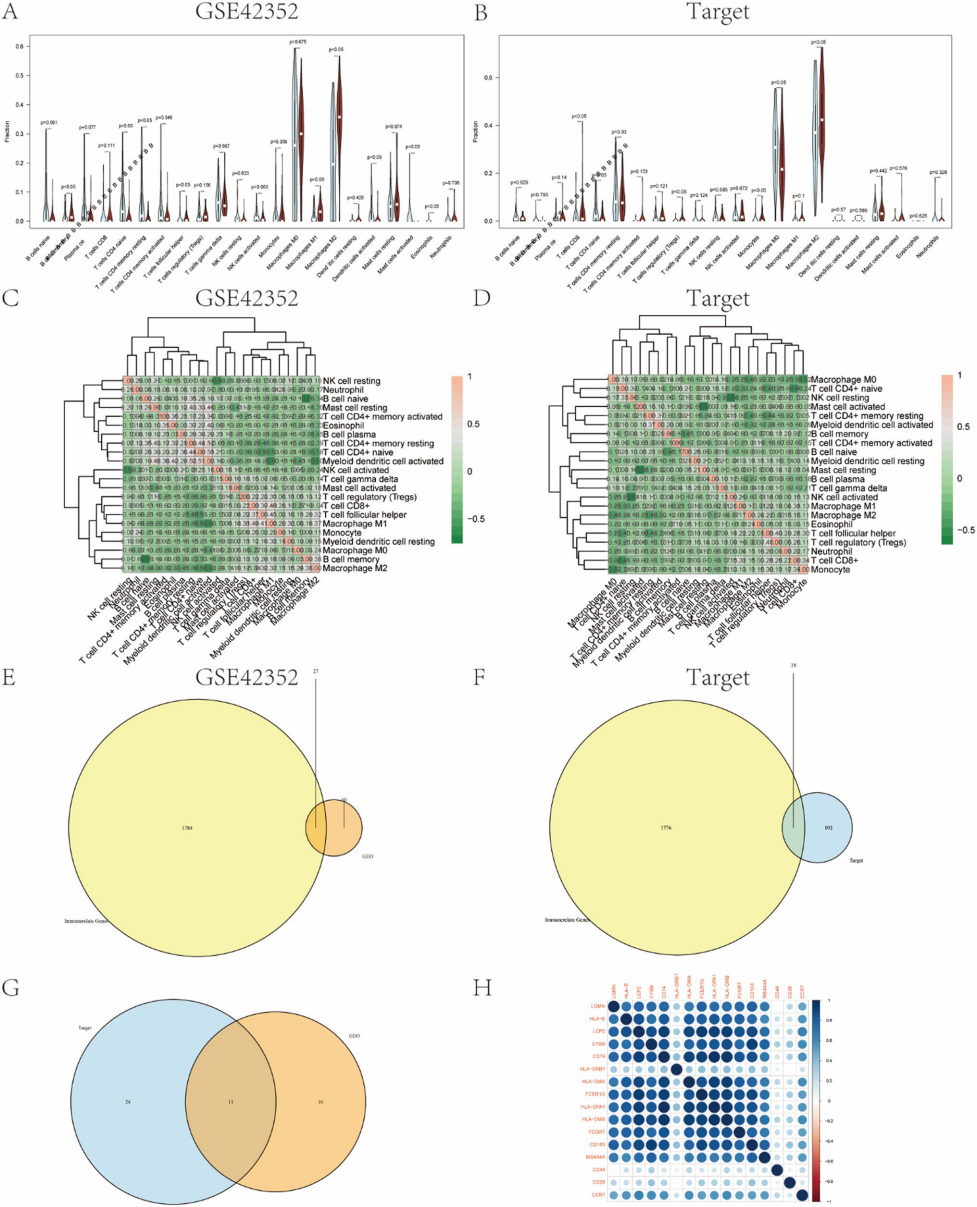
Correlation analysis of infiltration of immune cells and immunophenotype. **A** CIBERSORT package was used to calculate the potential difference in infiltration expression of 22 kinds of immune cells in 103 osteosarcoma samples derived from the test set using LM22 algorithm. The figure displays the differences in infiltration expression of 22 types of infiltrating immune cells between high and low expression groups of LGMN, with violin plots illustrating the expression differences of each immune cell type between the groups. Blue represents the low expression group, red represents the high expression group, and P value represents the significant difference with p < 0.05 being significantly different. **B** Verification of the difference in infiltration expression of 22 immune cells in 101 osteosarcoma samples between the high and low LGMN expression groups. The violin plot of blue represents the low expression group and red represents the high expression group, and the P value represents the significant difference with p < 0.05 being significantly different. **C** Correlation heat plot of 22 immune cells in the test set. **D** Correlation heat plot of 22 immune cells in the test set. **E** Venn diagram of the intersection of DEGs and immune-related genes in the test set. **F** Venn diagram of the intersection of DEGs and immune-related genes in the test set. **G** Venn diagram of the intersection of immune-related DEGs in the test set and the validation set. **H** Heat plot of immune-related DEGs and expression of immune cell surface markers

The immune cell infiltration was next examined. We used CIBERSORT package and LM22 algorithm to compare the two LGMN expression groups in terms of their infiltrating abundance of 22 types of immune cells, which included T cells, B cells, plasma cells, natural killer cells, and various myeloid subsets, etc. Moreover, we compared the two LGMN expression groups regarding their infiltration expression of infiltrating immune cells of 22 types in the test and validation sets. Thereafter, we drew violin plots and correlation heatmaps to show the potential differences between the two groups in the abundance of each immune cell (Fig. 7A–D). The above analysis of the immune infiltration indicated significant differences in the expression of Native CD4 + T cells and M2 macrophages between the high and low LGMN expression groups in the test set.

We obtained a list of immune-related genes from the ImmPort database, a list of intersections the DEGs from the test set (Fig. 7E) and a list from the verification set (Fig. 7F) based on the list of phenotyping-related genes. Then we drew a VennDiagram using the VennDiagram package. The immune-related DEGs of the data set were intersected, from which, 11 immune-related DEGs were finally obtained (Fig. 7G). Thereafter, based on the immune-infiltration analysis, we examined the possible correlation among these various phenotyptic-related genes and the expressions of Native CD4 + T cells and M2 macrophages surface markers in the test set. The heat plot package in R language was used to visualize the highly correlated results to display the correlation coefficient and to construct the correlation heatmap (Fig. 7H). Despite the relatively small number, our deconvolution method identified these genes among over 1700 immune-related genes, suggesting their potential significant roles in immune infiltration, particularly with notable associations to Native CD4 + T cell and M2 macrophage infiltration.

The above results indicated that Native CD4 + T cells and M2 macrophages were significantly involved in the immune microenvironment of osteosarcoma. More importantly, the pathogenesis and development of osteosarcoma might also be related to inflammatory and metabolic pathways of the bone.

### Evaluation of diagnostic performance and survival analysis of LGMN molecule for osteosarcoma

Finally, to evaluate the diagnostic performance of LGMN for cancer, we drew a receiver operating characteristic (ROC) curve by using the pROC package to determine whether LGMN expression could enable to distinguish the 15 normal samples from the 103 tumor samples in the test set. The optimal cut-off value of the highest likelihood ratio was determined to determine the recognition threshold of osteosarcoma by LGMN. The value of 0.799 obtained from the area under the curve (AUC) indicated that LGMN expression exhibited an excellent diagnostic value for osteosarcoma (Fig. 1B). We continued to use pROC packages to evaluate the overall survival rate of each biomarker in the subsequent risk model.

To further analyze effect of LGMN expression on the prognosis of osteosarcoma, we employed a survival package to compare the prognosis of the validation sets in terms of overall survival (OS), progression-free survival (PFS) and time to progression (TTP). We found a significant difference in the three values between OS (Fig. 1C) and DFS (Fig. 1D).

### Cox regression analysis and risk score

**Fig. 8:**
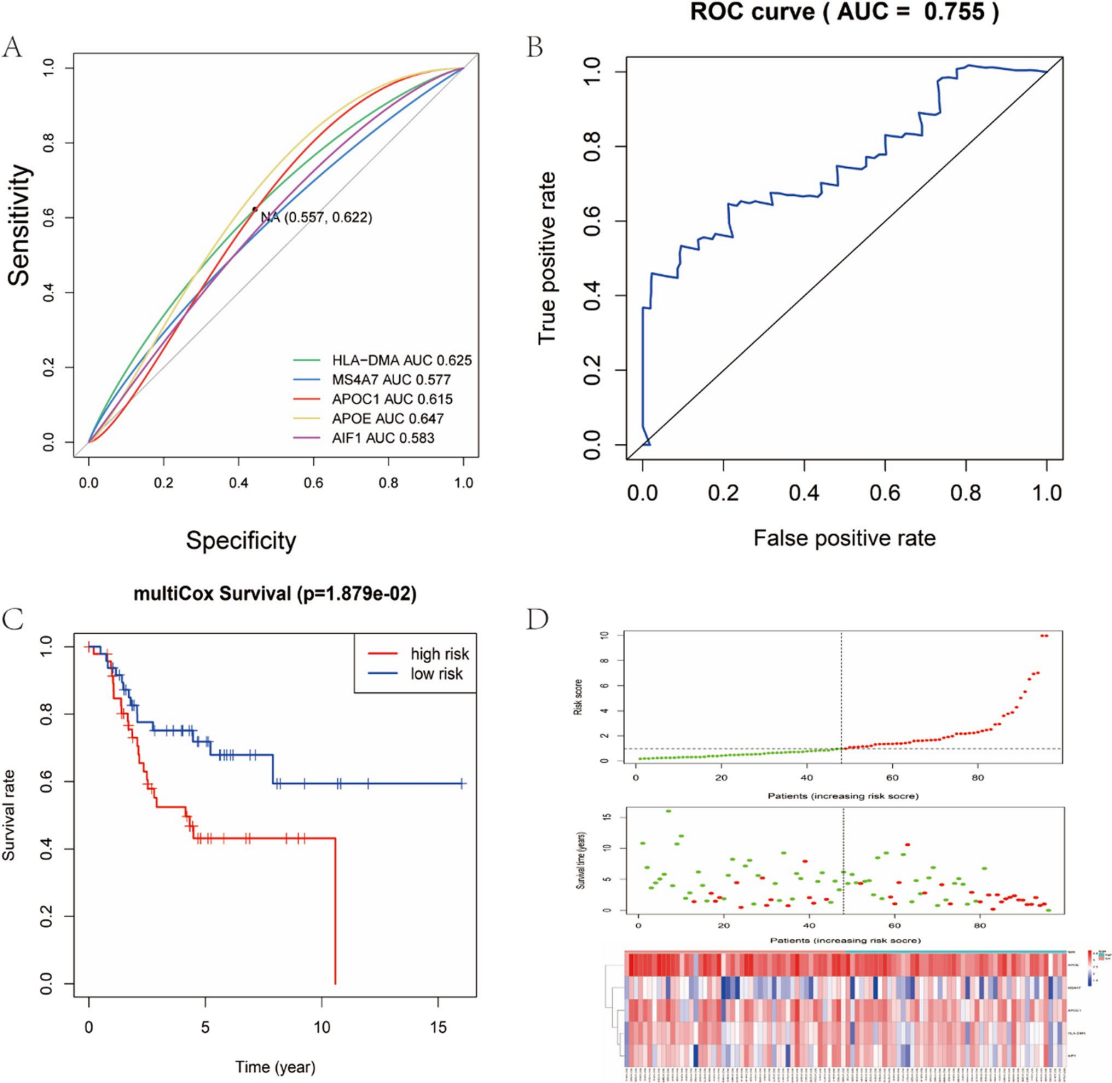
Cox regression analysis and risk assessment analysis. **A** ROC curve of the 5 distinct gene sets (HLA-DMA, MS4A7, APOC1, APOE, A1F1) to evaluate the total survival prognosis of each gene set in the TARGET database. **B** ROC curve of the 5 gene model risk score to evaluate the total survival prognosis. **C** The effect of 5 gene set as a potential risk model for the prognosis of OS was statistically significant. **B** ROC curve verified the accuracy of 4 gene sets and models in predicting both survival and prognosis of osteosarcoma. **D** The risk curve (top), survival status (middle) and risk heat plot (bottom) of the selected gene set

We next performed univariate Cox regression analysis of the gene matrix of TARGET database, extracted the results of univariate regression analysis of 26 common DEGs and conducted further multivariate Cox regression analysis of this identified gene set. Finally, *HLA-DMA*, *MS4A7*, *APOC1*, *APOE* and *AIF1* were found to be significantly altered (P < 0.05) (Table 3). The results indicated that the four DEGs were significantly correlated and can exert significant impact on the prognosis of osteosarcoma, and thus can be regarded as potential high-risk biomarkers for osteosarcoma (Fig. 8A).

**Table 3:** List of risk models for multivariate Cox regression analysis

id	coef	exp(coef)	se(coef)	z	Pr( >|z|)
AIF1	0.8440	2.3256	0.4028	2.0953	0.0361
MS4A7	0.6617	1.9381	0.3164	2.0912	0.0365
APOC1	0.5274	1.6946	0.2468	2.1373	0.0326
APOE	− 0.6373	0.5287	0.2451	− 2.5998	0.0093
HLA-DMA	− 1.3107	0.2696	0.4961	− 2.6422	0.0082

To further explore the prognostic value of the above five identified sets of genes for osteosarcoma, we analyzed the overall survival (OS) of the low and high risk groups with the survival kit as a risk factor in the clinical survival data of the TARGET database (Fig. 8C). Then, we used the survival ROC package to verify the accuracy of this gene set and evaluated the model to predict the prognosis of osteosarcoma. The value of 0.755 obtained from the comparison indicated that the gene set was highly accurate in predicting the overall survival of osteosarcoma (Fig. 8B). Subsequently, we plotted the risk curve (top), survival status (middle), and risk heatmap (bottom) for this set of genes (Fig. 8D).

### LGMN and risk model mutation analysis

**Fig. 9:**
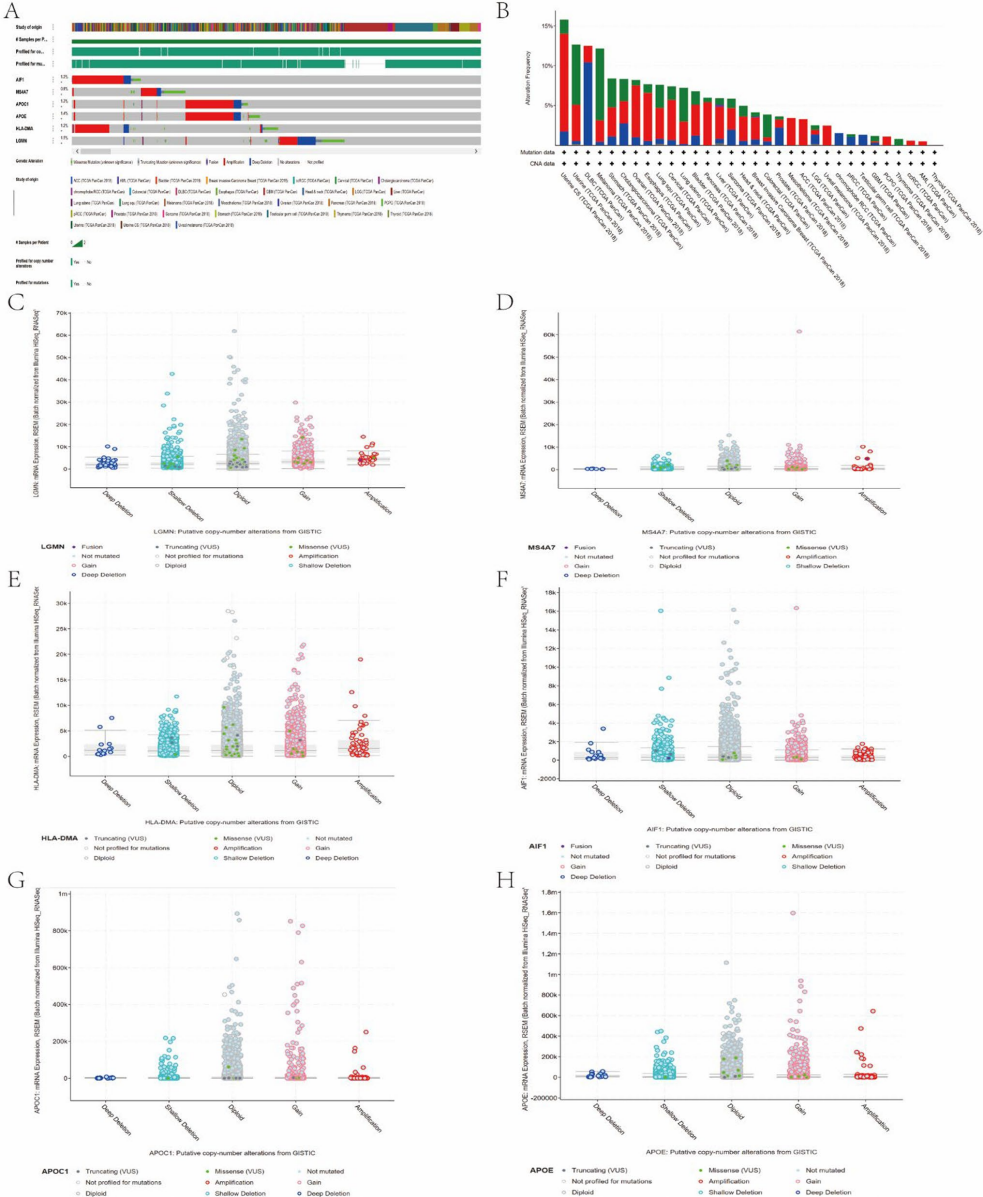
Mutational analysis of LGMN in pancancerous species. **A** cBioPortal database was used to analyze the distribution probability proportion of LGMN and 5 gene risk models in 32 TCGA pan-cancer data sets. **B** A Schematic diagram of LGMN mutation type analyzed by risk model of LGMN and 5 distinct genes in cBioPortal database. **C**–**H** CNVs COPY number variation analysis for each gene: LGMN (**C**), MS4A7 (**D**), HLA-DMA (**E**), A1F1 (**F**), APOC1 (**G**), APOE (**H**)

Since this study demonstrated that LGMN was directly related to the prognosis of osteosarcoma, we conducted an in-depth analysis of this genetic mutation. We analyzed the distribution of the various mutation types (Fig. 9A) and mutation status (Fig. 9B) of the five gene risk models in 32 TCGA pan-cancer data sets using cBioPortal database (Table 4). We found that most of the genes showed amplified mutations. Figure 9C–H shows the variation in CNVs copy number of each gene.

**Table 4:** Correlation list of risk model gene set mutations obtained from cBioPortal network platform (OR > 1 indicates that the gene aggregation mutation has a high risk in the pan-cancerous species)

Gene A	Gene B	Not Count	Odds Ratio	p-Value	q-Value
APOC1	APOE	122	> 3	< 0.001	< 0.001
AIF1	HLA-DMA	86	> 3	< 0.001	< 0.001
AIF1	MS4A7	9	2.922	< 0.001	< 0.001
HLA-DMA	LGMN	10	2.586	< 0.001	< 0.001
MS4A7	HLA-DMA	7	2.632	< 0.001	< 0.001
APOE	LGMN	9	2.195	< 0.001	< 0.001
MS4A7	LGMN	7	2.388	< 0.001	0.001
AIF1	APOC1	6	1.772	0.011	0.019
MS4A7	APOC1	5	1.989	0.011	0.019

### Construction and correlation analysis of osteosarcoma subtypes

**Fig. 10:**
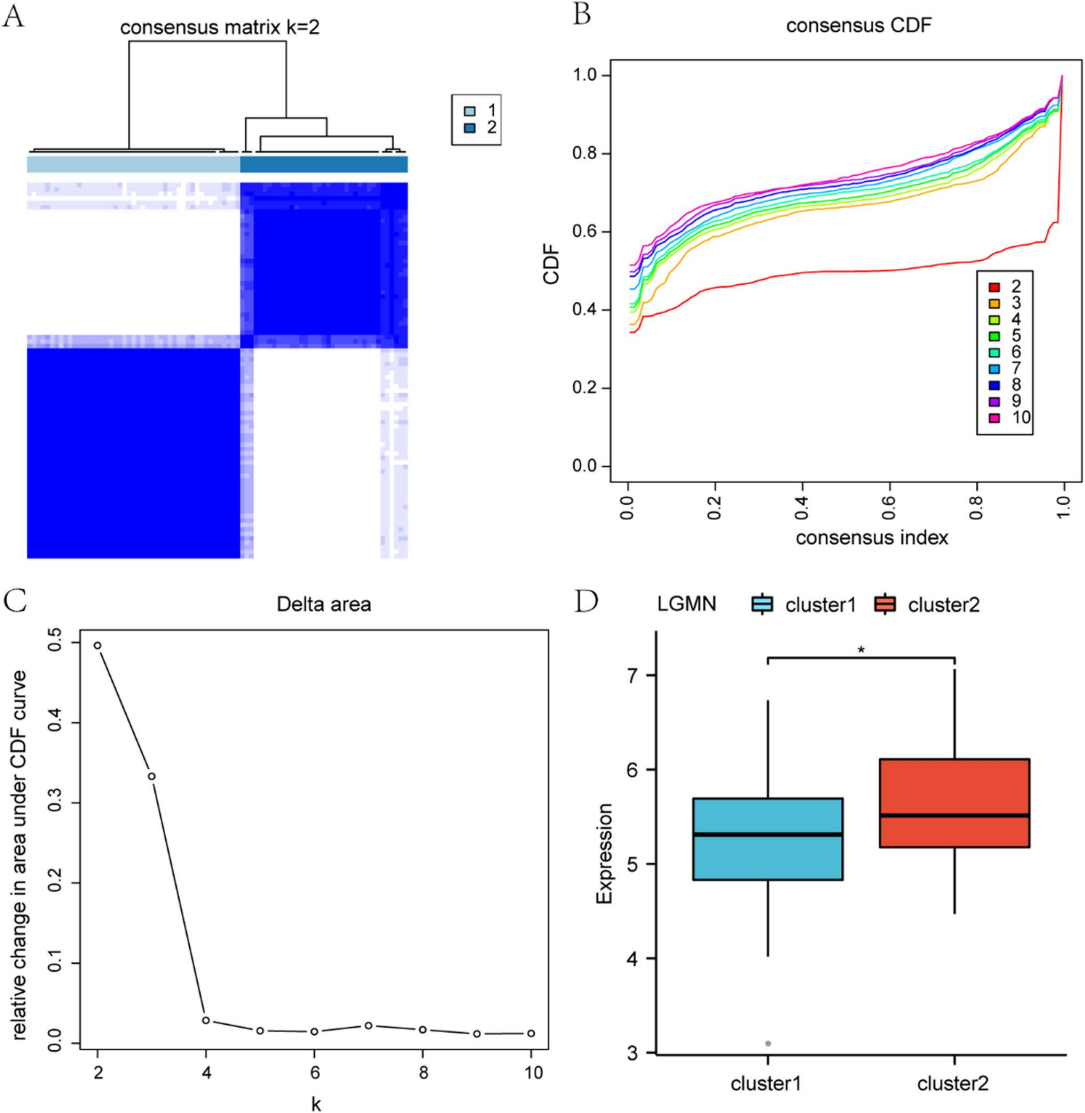
Consensus clustering analysis. **A** Figure displaying the consensus clustering results of osteosarcoma (OS) samples. **B** Plot of the Consensus Cumulative Distribution Function (CDF) from the consistency clustering analysis. **C** Consensus Matrix plot derived from the consistency clustering analysis. **D** Comparative illustration of LGMN expression across different cancer subtypes in osteosarcoma (OS), with results presented in a grouped comparison chart. OS refers to Osteosarcoma, CDF to Empirical Cumulative Distribution Function; Cluster1 is depicted in blue, and Cluster2 in red

To explore the cancer subtypes within the osteosarcoma (OS) samples in The Cancer Genome Atlas (TCGA-OS) dataset, we utilized the R package ConsensusClusterPlus. Based on the expression levels of the target gene LGMN in the OS samples from the TCGA-OS dataset, consensus clustering analysis was employed to identify distinct cancer subtypes related to osteosarcoma. As a result, two osteosarcoma subtypes were determined: Cluster 1 and Cluster 2 (Fig. 10A–C). The findings indicated significant differences between these two osteosarcoma subtypes.

Subsequently, we further analyzed the variations of the target gene LGMN among the different cancer subtypes of osteosarcoma. The results showed that the target gene LGMN exhibited statistically significant differences among the various cancer subtypes of osteosarcoma (P < 0.05) (Fig. 10D).

## Discussion

As the most common primary bone-borne malignancy [28] in children and adolescents, osteosarcoma, little significant progress has been made in prognosis of its patients over the past 30 years. Besides, its 5-year survival with lung metastasis remains as low as 15% [29]. A number of different factors have been reported to affect its prognosis, which include age, tumor volume, tumor location, surgical resection margin, histological response of preoperative chemotherapy, minimal recurrence interval, to name just a few [30]. However, due to limited understanding of its pathogenesis and lack of effective therapeutic targets and treatment strategies, several problems still exist with the recurrence, metastasis and emergence of multi-drug resistance during the management of this malignancy. These problems entail potential biomarkers to be identified for accurate diagnosis, efficacious treatment and good prognosis. Fortunately, techniques such as microarray and high-throughput sequencing facilitate to explore possible genetic changes in tumors. They have been widely used to predict the potential targets for treating many cancers, including osteosarcoma. In this study, we have performed a comprehensive analysis of the microarray datasets and discovered that LGMN expression could increase the incidence of osteosarcoma by potentiating pathways. The results of this study have corroborated that the prognosis of cancer patients could be related to LGMN expression, which suggest that LGMN expression can be used as a novel biomarker in predicting prognosis of cancer patients.

Using the GSE42352 dataset, we found significant differences in LGMN expression between osteosarcoma and the normal samples. We also verified the high expression of LGMN in the TARGET-OS dataset. Interestingly, increased expression and tumor promoting function of LGMN have been reported in several carcinomas previously such as cervical cancer [31], gastric cancer [32], oral cancer [33], breast cancer [34], neuroblastoma [35], pancreatic cancer [36], ovarian cancer [37] and melanoma [38, 39]. Thus, LGMN is assumed by this study as pivotal protein that is significantly involved in the regulation of angiogenesis, invasion and metastasis of tumors [40, 41]. In summary, substantial LGMN expression in various cancer tissues rather than in normal tissues can make it an ideal target for cancer treatment [42]. To our knowledge, there has been scarcely any research previously related to the diagnostic and prognostic values of LGMN expression. Against this background, the present study has been conducted to investigate the potential prognostic value of LGMN expression for osteosarcoma.

LGMN has been reported to exhibit good diagnostic value by mapping the subjects' working characteristic (ROC) curves onto prognostic values of survival prediction for osteosarcoma. For instance, a recent meta-analysis [43] showed that LGMN was overexpressed in cancer compared to normal tissues and its level was higher in phase III–IV diseases than phase I–II diseases. In addition, LGMN overexpression was found to be associated with the poor prognosis and clinical staging of different cancers [20, 44]. For example, in patients with rectal cancer, cases with overexpressed LGMN displayed lower overall survival rate than cases with low LGMN expression. Moreover, in another example, a study on glucose dependent melanoma found that high LGMN expression was associated primarily with local invasion by the tumor. The overall survival rate of these patients was poor [38]. LGMN expression is also considered as an independent prognostic factor for the overall survival rate of patients with gastric cancer and increased LGMN expression was significantly associated with peritoneal metastasis [45]. In addition, as high LGMN expression was also linked with a poor prognosis of in situ cancer within the breast duct, where it may act as a potential biomarker for predicting the development of in situ cancer within the breast duct into an invasive disease [46]. Since LGMN appears to be intimately involved in the development and progression of tumors, it is possible that it can function as a biomarker for monitoring, diagnosis, therapeutic targets, and prognosis of tumors.

In this study, the results of GO, KEGG and GSEA enrichment analyses suggested that the development of osteosarcoma, infection immunity and high LGMN expression might be related to cancer and immune microenvironment. In addition, LGMN expression in the normal cells has been found to be significantly low [43, 47] compared to that in the tumor cells or tumor-associated macrophages (TAMs). In light of the above findings, we further explored LGMN expression’s role in the immune system of osteosarcoma. The results of immune-infiltration and immune-phenotype correlation analyses also revealed an essential role of native CD4 T cells and M2 macrophages in the immune microenvironment of osteosarcoma. These findings are essential to identify the proteins involved in tumor cells and TAMs because the results can be used as a biomarker for the prognosis of osteosarcoma. The present study also suggested that it is highly likely that LGMN expression might be used as a biomarker if LGMN expressed highly in osteosarcoma and tumor-associated macrophages (TAMs). Therefore, it is necessary to explore the potential role of TAMs in osteosarcoma further. Interestingly, it was found that upregulation in LGMN expression in the macrophages promoted proliferation and angiogenesis of the cancer cells under both in vitro and in vivo settings [32]. A number of recent studies have demonstrated that LGMN acts as an "upstream" activator of the CTSL-C3-IFN-axis in the human CD4 (+) T cells and is thus considered as an essential proponent of human Th1-induced tumors [48]. In addition, studies on the pancreatic cancer have suggested that LGMN expression may play a key role [36] in the immune escape by dendritic cells treated with PEX. For instance, some studies have shown that the tumor microenvironment could be directly associated with the invasion, metastasis, drug resistance and invasion of the various tumor cells, including natural and acquired immune cells. Hence, because of TAMs’ critical role in the aggressive behavior and metastatic ability of the tumor cells, it is crucial to inhibit TAMs and tumor cells to achieve optimal effects of the pharmacological drugs [49]. Thus, it seems evident that LGMN can serve as an ideal biomarker as for the therapeutic agents directly targeted toward tumor cells. It has been also found that LGMN-based DNA vaccine can alleviate the activity [50] of macrophages in the different metastatic cancers such as cancers of the breast, the colon and the lung. This vaccine can effectively promote activation of dendritic cells and co-activation of cytotoxic CD8 T cells, which is also accompanied by mitigation of macrophage-expressing LGMN. Therefore, development of innovative immunotherapy strategies for targeting LGMN-mediated expression of macrophages or other immune cell activities may help to alleviate metastasis and angiogenesis.

Our study capitalizes on sophisticated bioinformatics tools to successfully delineate two distinct subtypes within osteosarcoma samples, an accomplishment underpinned by a profound understanding of LGMN expression patterns in the TCGA-OS dataset. The distinction between Cluster 1 and Cluster 2 not only underscores the heterogeneity intrinsic to osteosarcoma but also introduces a new dimension to its molecular classification. Of particular importance, the differential expression of LGMN observed between these subtypes implies its potential as a biomarker for subgroup differentiation, thereby furnishing a robust molecular basis for the implementation of personalized medicine strategies in osteosarcoma.

Our study has highlighted the crucial role of immune cell infiltration and LGMN expression in the osteosarcoma microenvironment, specifically in immune evasion and patient prognosis. LGMN-positive tumor-associated macrophages (TAMs) are believed to be key players in creating an immunosuppressive environment, aligning with Li’s [51] work on the immunomodulatory role of extracellular vesicles (EVs) in osteosarcoma. Moreover, Li et al. [52] have shown the significance of the miR-339-3p/IGF1R axis in osteosarcoma's resistance to cisplatin, potentially linked to immune cell infiltration. Guo et al. [53] have further explained the impact of exosomal circular RNAs on chemoresistance, which may intersect with the immunosuppressive functions of LGMN TAMs. Additionally, Li, Kang et al. [54] have proposed innovative therapeutic approaches targeting the tumor and bone microenvironment, with potential implications for osteosarcoma treatment. The exploration of TIMs, TAMs, and PS-antibody targeting in cancer immunotherapy by Dayoub and Brekken [55] could offer valuable insights for immunological interventions in osteosarcoma. Nishida [56] has also shed light on oncogenic pathways influencing the immunosuppressive microenvironment in liver cancer, providing a comparative perspective that enhances our understanding of similar environments in osteosarcoma. These studies contribute to our understanding of how immune cell infiltration and LGMN expression interact to impact immune evasion in the osteosarcoma microenvironment. The potential significance of LGMN TAMs in this process is emphasized, providing a new avenue for future research and therapeutic developments in osteosarcoma.

There are some limitations associated with this study that should be considered when interpreting the results. First, due to the small sample size, it is necessary to further validate the results in larger cohort of patients. Moreover, the pathway analysis conducted by GO, KEGG, GSEA, and interaction between the factors should be investigated further in detail. Second, due to the limited availability of osteosarcoma samples, which is vital data, it is important to study osteosarcoma using multiple datasets rather than one. However, this study is mainly dependent on the batch effects due to the lack of various statistical controls. Third and finally, further experimental evidence for immunogenomic analysis is needed to completely elucidate the role of the enriched pathways in the regulation of immune microenvironment.

## Conclusion

Our investigation reinforces the pivotal role of LGMN as a prognostic indicator in osteosarcoma, shedding light on its integral function in modulating the tumor's immune landscape. The recognition of distinct LGMN-expression-based subtypes via consensus clustering constitutes a pivotal step towards precision oncology, underlining LGMN's promise as both a therapeutic intervention point and a classifier for risk categorization.

## Data Availability

Data are available in a public, open-access repository. Data are available on reasonable request. All data relevant to the study are included in the article. The GSE42352 dataset can be accessed in the GEO database (https://www.ncbi.nlm.nih.gov/geo/query/acc.cgi?acc=GSE42352); the Target-OS dataset refers to the data originating from the TARGET project (https://ocg.cancer.gov/programs/target/data-matrix).
